# Regulation of NMDA Receptor Signaling at Single Synapses by Human Anti-NMDA Receptor Antibodies

**DOI:** 10.3389/fnmol.2022.940005

**Published:** 2022-07-28

**Authors:** Charles A. Dean, Sarah R. Metzbower, Scott K. Dessain, Thomas A. Blanpied, David R. Benavides

**Affiliations:** ^1^Department of Neurology, University of Maryland School of Medicine, Baltimore, MD, United States; ^2^Department of Physiology, University of Maryland School of Medicine, Baltimore, MD, United States; ^3^Lankenau Institute for Medical Research, Wynnewood, PA, United States

**Keywords:** autoimmune encephalitis, calcium signaling, synaptic, NMDA receptor, neurotransmission

## Abstract

The NMDA receptor (NMDAR) subunit GluN1 is critical for receptor function and plays a pivotal role in synaptic plasticity. Mounting evidence has shown that pathogenic autoantibody targeting of the GluN1 subunit of NMDARs, as in anti-NMDAR encephalitis, leads to altered NMDAR trafficking and synaptic localization. However, the underlying signaling pathways affected by antibodies targeting the NMDAR remain to be fully delineated. It remains unclear whether patient antibodies influence synaptic transmission *via* direct effects on NMDAR channel function. Here, we show using short-term incubation that GluN1 antibodies derived from patients with anti-NMDAR encephalitis label synapses in mature hippocampal primary neuron culture. Miniature spontaneous calcium transients (mSCaTs) mediated *via* NMDARs at synaptic spines are not altered in pathogenic GluN1 antibody exposed conditions. Unexpectedly, spine-based and cell-based analyses yielded distinct results. In addition, we show that calcium does not accumulate in neuronal spines following brief exposure to pathogenic GluN1 antibodies. Together, these findings show that pathogenic antibodies targeting NMDARs, under these specific conditions, do not alter synaptic calcium influx following neurotransmitter release. This represents a novel investigation of the molecular effects of anti-NMDAR antibodies associated with autoimmune encephalitis.

## Introduction

The molecular correlates of learning and memory in the central nervous system are encoded at synapses, the fundamental unit of information transfer between neurons. Experience drives alterations in synaptic transmission in a process termed synaptic plasticity, whereby transmission between neurons is strengthened or weakened. At excitatory glutamatergic synapses, synaptic strength is tuned by alterations to the number or single-channel conductance of ionotropic glutamate receptors, primarily α-amino-hydroxy-5-methyl-4-isoxazolepropionic acid receptors (AMPARs). Another ionotropic glutamate receptor, the N-methyl-D-aspartate receptor (NMDAR), governs this plasticity through its Ca^2+^ permeability and control of postsynaptic signaling. The level of Ca^2+^ influx through NMDARs at the postsynaptic membrane controls NMDAR-dependent plasticity (Sabatini et al., 2002; Sanderson and Dell’Acqua, 2011; Lisman et al., 2012). Furthermore, recent evidence suggests that spontaneous neurotransmitter release events modulate synaptic tuning (Andreae and Burrone, 2014, 2018; Kavalali, 2015).

Anti-NMDAR encephalitis is an autoimmune disease whereby autoantibodies target the NMDAR (Dalmau et al., 2008). This disease is marked by neuropsychiatric symptoms including memory deficits, psychosis, catatonia, hyperkinetic movement disorder, language dysfunction, seizures, and autonomic dysfunction (Dalmau et al., 2008; Graus et al., 2016; Dalmau and Graus, 2018). Coma and death can occur in severe cases. Although this disease was relatively recently described, much work has defined the pathophysiological effects of anti-NMDAR antibodies derived from patient biosamples. Autoantibodies target a common epitope within the obligate GluN1 subunit of NMDARs, within the N-terminal domain (NTD), and cause internalization of NMDARs that is dependent on receptor crosslinking (Dalmau et al., 2008; Dalmau and Graus, 2018). The main epitope has been mapped to the N368/G369 region of the extracellular NTD of GluN1 (Gleichman et al., 2012). As anti-GluN1 antibodies decrease NMDAR surface expression through crosslinking and internalization of receptors (Dalmau et al., 2008; Hughes et al., 2010), the prevailing hypothesis is that patient symptoms arise from loss of synaptic NMDARs (Mikasova et al., 2012; Moscato et al., 2014; Planagumà et al., 2015, 2016; Dalmau and Graus, 2018).

Current evidence suggests that after exposure to pathogenic GluN1 antibodies, internalization of NMDARs occurs on a timescale of hours (Moscato et al., 2014). However, there is a paucity of information on pathogenic GluN1 antibody effects at short timepoints, and it is critical that we understand this for two reasons. First, it is important to define fully whether antibody binding disrupts the function of NMDARs at native synapses and contributes to the pathophysiology of anti-NMDAR encephalitis. Secondly, antibodies may have effects on NMDARs in addition to cross-linking and internalization. Indeed, evidence suggests that a population of NMDARs remain on the surface and at the synapse even at later timepoints following pathogenic antibody exposure (Moscato et al., 2014; Ladépêche et al., 2018), potentially with altered functionality caused by interaction with GluN1 antibodies. Furthermore, metabotropic NMDAR function has been established in synaptic plasticity (Nabavi et al., 2013; Aow et al., 2015; Dore et al., 2015) and remains unexplored in the molecular pathophysiology of anti-NMDAR encephalitis. These gaps in knowledge have made development of targeted therapeutics difficult, and current therapies do not target the underlying pathology. Defining further the pathogenic effects of GluN1 antibodies on NMDAR function will contribute to our understanding of how function of surface NMDARs may be disrupted, even at later stages of antibody exposure and disease, and provide insights into novel therapeutic targets for anti-NMDAR encephalitis.

We have previously shown that human monoclonal GluN1 antibodies, derived from a patient with anti-NMDAR encephalitis, recapitulate key cellular (Sharma et al., 2018a), physiological (Taraschenko et al., 2021b), and behavioral features (Sharma et al., 2018a; Taraschenko et al., 2021a) of the disease. We hypothesized that short-term GluN1 antibody exposure disrupts synaptic NMDAR function. Here, we demonstrate critical functions of synaptic NMDARs are not disrupted by GluN1 antibodies using a combination of immunohistochemical and live-cell imaging techniques in neuron cultures.

## Materials and Methods

### Animals

All experiments were performed with approval from the Institutional Animal Care and Use Committee of University of Maryland School of Medicine. Experiments were performed using mixed-sex neuron cultures derived from Sprague-Dawley rat embryos (Charles River Laboratories, Wilmington, MA, United States). All animal procedures were performed in accordance with the University of Maryland Baltimore animal care and use committee’s regulations.

### Human Monoclonal Antibodies

Human monoclonal GluN1 antibodies specific for GluN1 were derived from an 18 year-old female patient with anti-NMDA receptor encephalitis who presented with emotional lability, paranoia, and temporal lobe seizures (Sharma et al., 2018a). We have previously confirmed specificity for NMDARs using transfected HEK 293 cells (Sharma et al., 2018a) as well as a cell line expressing the GluN1-NTD (Sharma et al., 2018b) for four human clones 5F5, 3C11, 1D1, and 2G6 (data not shown). The 5F5 GluN1 human monoclonal antibody (GluN1 mAb) was previously shown to target GluN1 and stain mature primary neurons (data not shown). The 6A control human monoclonal antibody against BoNT serotype A (BoNT/A) heavy chain was isolated and cloned using the same hybridoma method (Adekar et al., 2008) and was previously confirmed to be non-reactive in brain and primary hippocampal neurons (Sharma et al., 2018a). The human monoclonal antibody 6A (Control mAb) was used as a control IgG for all experiments.

### Rat Primary Hippocampal Neuron Culture

Hippocampi were isolated from male and female embryonic day 18 Sprague-Dawley rat embryos, dissociated, and plated on glass coverslips coated with poly-L-lysine in plating media (Neurobasal medium supplemented with 2% B27, 2 mM Glutamax, 50 U/mL Penicillin-Streptomycin, and 5% bovine serum). Neurons were seeded at 50 k cells/well in 12-well plates. At 4 days *in vitro* (DIV 4), neurons were treated with 2 mM (+)-5-fluor-2’-deoxyuridine for 24 h, followed by exchange of growth media (Neurobasal medium supplemented with 2% B27, 2 mM Glutamax, 50 U/mL Penicillin-Streptomycin). Every 3–4 days thereafter, half of the culture medium was changed with fresh growth media until DIV indicated. All experiments were conducted no sooner than 48 h from last media change. Immunocytochemistry and Ca^2+^ imaging experiments were performed from DIV 18 to DIV 23.

### Immunocytochemistry

Neurons on coverslips were fixed in 4% paraformaldehyde (PFA) and 4% sucrose in PBS at room temperature (RT) for 10 min. Coverslips were then washed in PBS with 100 mM glycine (PBS-G) and permeabilized by incubation in PBS-G with 0.3% Triton X-100 (TX-100) for 20 min at RT. Neurons were then blocked for 20 min at RT in blocking buffer consisting of PBS-G supplemented with 5% donkey serum, 5% bovine serum albumin, and 0.1% TX-100. Primary antibodies were diluted in blocking buffer and incubated on coverslips overnight at 4°C. Neurons were washed with PBS-G after primary antibody incubation, then conjugated secondary antibodies diluted in PBS-G were applied for 1 h at RT. Coverslips were then washed with PBS-G and mounted with ProLong Glass Antifade mountant (Thermo Fisher Scientific, Waltham, MA, United States).

### Calcium Imaging Experiments

**FIGURE 1:**
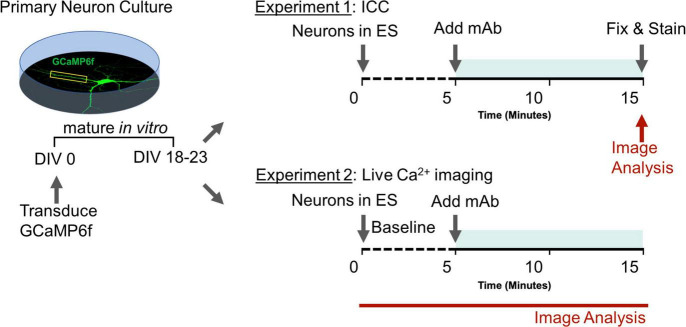
Experimental design to assess role of human GluN1 monoclonal antibodies on synaptic function. Teal shaded bar represents mAb incubation period. Red indicates imaging sessions. DIV, days *in vitro*; ES, extracellular solution; ICC, immunocytochemistry; mAb, human monoclonal antibodies.

Dissociated hippocampal neurons were prepared as described above, except that 25 k uninfected cells were plated with 20 k cells infected with pAAV.CAG.GCaMP6f.WPRE.SV40 (Penn Vector Core) on DIV 0 on each individual coverslip. All experiments were performed on a wide-field Nikon Ti2 using a 40×/1.3 NA oil-immersion objective and a Zyla 4.2 sCMOS camera. Time-lapse images were acquired at 20 Hz under continuous autofocus. An objective-heater maintained bath solutions at 37°C. Basal extracellular solution (ES) contained 0 mM Mg^2+^, 139 mM NaCl, 2.5 mM KCl, 1.5 mM CaCl2, 10 mM glucose, and 10 mM HEPES with pH adjusted to 7.4 with NaOH. Ca^2+^ imaging of miniature spontaneous Ca^2+^ transients (mSCaTs) was performed essentially as described previously (Metzbower et al., 2019) with several modifications. Neurons were removed from Neurobasal growth media and briefly rinsed in ES containing 1 μM TTX (Enzo, Farmingdale, NY, United States). Then, to isolate pharmacologically NMDAR-dependent mSCaTs, neurons were pre-incubated for 20 min in mSCaT imaging solution (MIS): ES supplemented with 1 μM TTX (Enzo), 10 μM DNQX (Sigma-Aldrich, St Louis, MO, United States), 20 μM ryanodine (Tocris Bioscience, Briston, United Kingdom), 1 μM thapsigargin (Sigma-Aldrich), and 5 μM nifedipine (Sigma-Aldrich). Baseline mSCaT activity was imaged for 5 min, after which human mAbs at 1 μg/mL were added to the bath solution and neurons were imaged for another 10 min. Processing and analysis of Ca^2+^ imaging data were performed as described previously (Metzbower et al., 2019). Briefly, regions of interest (ROIs) were drawn around single dendritic spines in MetaMorph (Molecular Devices, San Jose, CA, United States). Using custom MATLAB scripts (MathWorks, Natick, MA, United States), *F*_*baseline*_ was then determined at each spine ROI by averaging background-subtracted mean intensity every 10 frames and within each minute of imaging identifying the lowest positive value. In some analyses, individual spine F values were normalized to F value obtained over 5 min baseline period to yield running F value over the course of the imaging session on per spine basis. For each frame of imaging ΔF/F was calculated by (*F_*frame*_ – F_*baseline*_)/F_*baseline*_*. To detect mSCaTs, ΔF/F at each spine over time was analyzed in Clampex (Molecular Devices), and a template search identified peaks and peak amplitude based on an average shape profile. Only spines that produced at least one mSCaT during baseline were selected for analysis, and amplitude and frequency were normalized to baseline values within each spine. Plots for cell and spine were constructed over time. Experimenters were blind to experimental condition during imaging, spine selection, image processing, and data analysis (Experimental design summarized in Figure 1).

### Live Labeling at Spines and Synapses and Fluorescent Microscopy

To confirm localization of human mAbs at spines, GCaMP6f-infected neurons underwent the mSCaT imaging process followed immediately by the staining procedure described above. Secondary anti-human antibodies were utilized. Images were acquired using an Andor Dragonfly spinning disk confocal, using 60×/1.45 NA objective and a sCMOS camera (Andor Zyla) giving a final pixel size of 108 nm. Quantification of human IgG intensity at spines was performed in FIJI (ImageJ) by measuring background-subtracted intensity at ROIs drawn around dendritic spines. Spines were identified by GCaMP6f signal used as a morphological marker.

### Antibodies

For immunocytochemistry, the following primary antibodies were used: pan-shank (6.25 μg/mL, 75-089, NeuroMab, Davis, CA, United States; RRID:AB_10672418). The following secondary antibodies were used: donkey anti-human Alexa647 (1:200, 709-605-149, Jackson Immuno Research Labs, West Grove, PA, United States; RRID:AB_22340578) and donkey anti-mouse CF555 (1:500, 20037, Biotium, Fremont, CA, United States; RRID:AB_10559035).

### Statistical Analysis

Statistical analysis was performed in Prism (GraphPad, San Diego, CA, United States). A Kruskal-Wallis test (non-parametric) followed by multiple comparisons with Dunn’s correction was conducted for immunohistochemistry with more than two groups. A Welch’s *t*-test was used to compare baseline (0–5 min) and post-treatment (10–15 min) normalized basal F values. A Welch’s ANOVA was used to compare normalized basal F values and mSCaT experiments with more than two groups. Results are reported as mean ± SEM. Whiskers represent 5–95%. All microscopy experiments were repeated at least three times. In all analyses, a *p*-value of less than 0.05 was considered statistically significant. Experimenters were blind to experimental condition during all imaging, image processing, and data analysis.

### Code Accessibility

All MATLAB code used for Ca^2+^ imaging analysis is available on request. All code was run on Windows 7 and Windows 10 operating systems.

## Results

### Human Monoclonal GluN1 Antibodies Rapidly Localize to Mature Native Synapses

**FIGURE 2:**
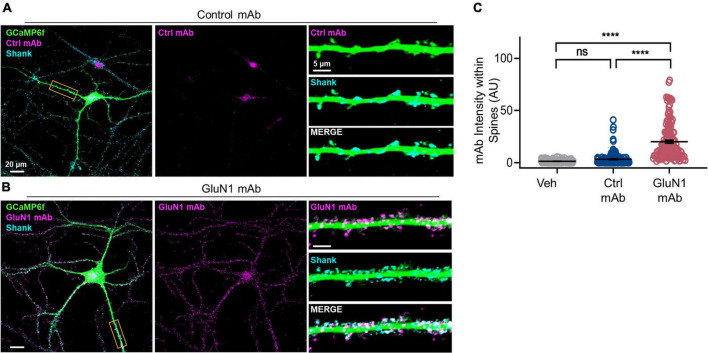
Human GluN1 monoclonal antibodies rapidly localize to spines in live hippocampal primary neuron culture. **(A)** Representative cultured hippocampal neuron transduced with GCaMP6f (green) and stained for human mAb (magenta) and synaptic marker Shank (cyan) following control mAb exposure. Right top, zoom in of boxed dendrite in left showing GCaMP6f and Ctrl mAb (magenta). Right middle, GCaMP6f and Shank (cyan). Right bottom, zoom in of GCaMP6f, human mAb, and Shank. Note lack of human control mAb labeling. **(B)** Representative hippocampal neuron, as in panel **(A)**, exposed to GluN1 mAb. Right bottom, note co-labeling of Shank with human GluN1 mAb (white). **(C)** Quantification of human mAb staining intensity at spines. A Kruskal-Wallis test with *post-hoc* Dunn’s revealed a statistically significant difference in mAb labeling between groups [*H*(3) = 240, *p* < 0.0001]. Control mAb labeling at spines was not significantly above background levels of vehicle (ns, *p* = 0.1) and mAb spine labeling was significantly different between GluN1 mAb and vehicle (*p* < 0.0001) and between GluN1 mAb and control mAb (*p* < 0.0001). Ctrl, control; mAb, human monoclonal antibody; ns, not significant; Veh, vehicle; ****p < 0.0001; ns, *p* > 0.05.

Our previous work in primary cultured neurons demonstrated that GluN1 mAb derived from a patient with anti-NMDAR encephalitis localizes to a subset of synapses after 45 min of incubation (Sharma et al., 2018a). Thus, this GluN1 mAb is well-suited to studies of individual monoclonal NMDAR-IgG antibodies and their effects on synaptic function at short time-points (Figure 1). After 45 min of GluN1 mAb incubation, however, significant internalization of the antibody had already occurred (Sharma et al., 2018a), and the initial pattern of mAb labeling at synapses is unknown. To test early GluN1 mAb labeling at native synapses, we conducted live labeling experiments in mature hippocampal primary neurons for brief, 10 min application times of human monoclonal antibodies (Figure 2). To confirm spine labeling, we utilized neurons expressing GCaMP6f as a morphology marker. Primary hippocampal neurons were matured to DIV 20 prior to staining. Following 10 min GluN1 mAb application to GCaMP6f-infected neurons, secondary antibodies were applied to detect human mAbs, and images were analyzed using confocal fluorescent imaging. The synaptic protein Shank was used as a marker of spines. The control mAb, 6A, exhibited no significant staining at dendritic spines (Figure 2A) relative to a vehicle-treated control conditions (images not shown; *p* = 0.1). Robust GluN1 labeling was visualized by GluN1 mAb (Figure 2B) at spines. Dunn’s Test for multiple comparisons revealed that the intensity of GluN1 mAb at spines was significantly higher than that in vehicle (*p* < 0.0001) and control mAb (*p* < 0.0001) conditions (Figure 2C). These data demonstrate that human GluN1 mAbs localize to dendritic spines within 10 min. Our observations of rapid labeling of monoclonal GluN1 antibodies to native synapses supports the potential for immediate effects of these pathogenic antibodies in the setting of anti-NMDAR encephalitis.

### Baseline Calcium Levels at Synaptic Spines Are Not Altered by Human Monoclonal GluN1 Antibodies

**FIGURE 3:**
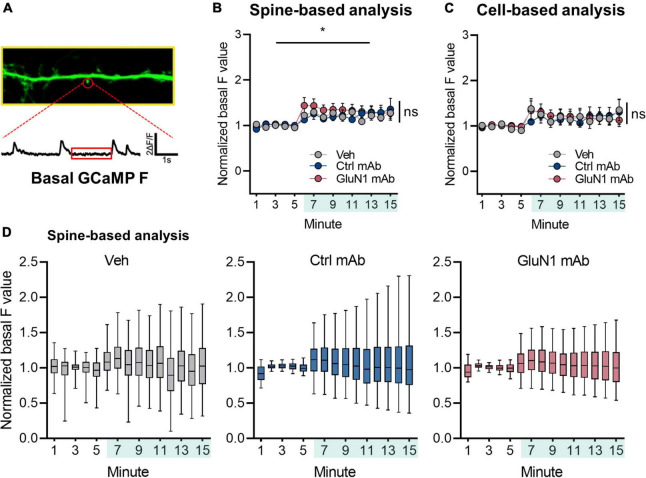
Basal Ca^2+^ at synaptic sites is not altered by human GluN1 antibodies. **(A)** Zoom in of dendrite of hippocampal cell transduced with GCaMP6f (green). Spine identified by red circle. Below, ΔF/F trace of spine region identified in panel **(A)** plotted against time. Red box indicates region analyzed as basal GCaMP Ca^2+^ measurement. **(B)** Normalized basal F trace of spine over recording session. Vehicle or mAb added after 5 min baseline; teal shaded bar in x-axis represents incubation period. A one-way Welch’s ANOVA was performed to compare the effect of mAb on normalized basal F values in final 5 min between groups. There was an effect of vehicle treatment (1.23 ± 0.076, *n* = 1,171 spines, *p* < 0.05, Welch’s *t*-test comparing baseline to final 5 min). There was no statistically significant difference between groups at final 5 min (control mAb 1.26 ± 0.16, *n* = 1,151 spines; GluN1 mAb 1.29 ± 0.12, *n* = 1,705 spines; one-way Welch’s ANOVA [*F*(2, 3,182) = 0.07, *p* = 0.9]. **(C)** Normalized basal F trace of spines analyzed on per cell basis. No statistically significant difference was observed on F values between treatments at final 5 min (control mAb 1.21 ± 0.46, *n* = 6 cells; GluN1 mAb 1.16 ± 0.33, *n* = 6 cells; Veh 1.25 ± 0.51, *n* = 7 cells; one-way Welch’s ANOVA [*F*(2, 10) = 0.06, *p* = 0.9]. **(D)** Box plots of normalized basal F values in individual treatments over time. Ctrl, control; mAb, human monoclonal antibody; ns, not significant; Veh, vehicle; *p < 0.05; ns, *p* > 0.05.

To determine if human monoclonal GluN1 antibodies alter Ca^2+^ handling at synaptic sites, we utilized the genetically encoded Ca^2+^ indicator GCaMP6f for live-cell imaging of NMDAR Ca^2+^ influx at synapses in cultured neurons, as previously reported (Andreae and Burrone, 2015; Metzbower et al., 2019). We measured basal F over time under conditions of blockade of neuronal activity, intracellular Ca^2+^ stores, and extracellular sources of Ca^2+^ (Figure 3A). The signal intensity of GCaMP6f was observed to vary slightly over the recording session, as has been observed in our prior work (Metzbower et al., 2019), and the baseline GCaMP6f signal increased significantly following addition of vehicle (1.23 ± 0.076, *n* = 1,171 spines, *p* < 0.05, Welch’s *t*-test comparing baseline to final 5 min; Figure 3B). However, we found no effect of human monoclonal antibodies on normalized basal F values compared to vehicle condition (control mAb 1.26 ± 0.16, *n* = 1,151 spines; GluN1 mAb 1.29 ± 0.12, *n* = 1,705 spines; [*F*(2, 2,427) = 0.092, *p* = 0.9; Figure 3B]. Similar results were observed when analyzed on per cell basis rather than per spine (Figure 3C). Overall, the median normalized basal F values remained stable throughout the recording session following addition of vehicle or mAbs when analyzed on per spine basis (Figure 3D), as well as per cell basis (Supplementary Figure 1). These findings support the notion that brief exposure to antibodies does not dramatically change Ca^2+^ mobilization at synaptic spines in mature primary culture. Further, it suggests the lack of rapid cellular toxicity from the brief exposure to antibodies in this experimental paradigm. However, little is known about NMDAR mediated Ca^2+^ at native synapses in response to human monoclonal GluN1 antibody exposure. We sought to evaluate this by measuring NMDAR-mediated mSCaTs.

### Human Anti-NMDAR Antibodies Have Limited Effect on the Amplitude or Frequency of NMDAR-Mediated Miniature Spontaneous Ca^2+^ Transients

To our knowledge, the only study that tested the effects of pathogenic anti-NMDAR antibodies on native NMDAR function at short timepoints found that cerebrospinal fluid (CSF) from patients prevented NMDAR-dependent long-term potentiation (LTP) at hippocampal CA3-CA1 synapses in acute slice after just 5 min of exposure (Zhang et al., 2012). This suggests that pathogenic GluN1 antibodies prevent normal synaptic NMDAR function at time points before significant antibody-mediated loss of synaptic receptors has been reported. The underlying mechanism for this, however, remains unexplored. Because LTP at these synapses requires Ca^2+^ influx through NMDARs, an attractive mechanism by which pathogenic GluN1 antibodies prevent normal plasticity is decreased Ca^2+^ influx at synaptic receptors.

**FIGURE 4:**
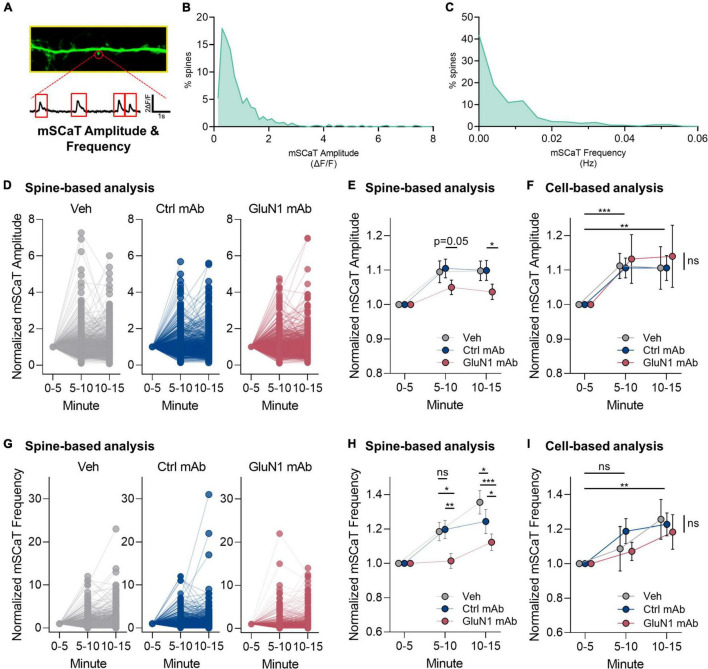
Human GluN1 mAbs have limited effect on NMDAR-dependent miniature spontaneous Ca^2+^ transients (mSCaTs). **(A)** Zoom in of dendrite of hippocampal cell transduced with GCaMP6f (green). Spine identified by red circle. Below, ΔF/F trace of spine region identified in panel **(A)** plotted against time. Red box indicates regions analyzed as mSCaTs. **(B)** Frequency histogram of mSCaT amplitude (ΔF/F) for individual synapses across 747 spines from 7 cells in vehicle condition. **(C)** Frequency histogram of mSCaT frequency (Hz) for individual synapses across 747 spines from 7 cells in vehicle condition. **(D)** Dot plots of normalized mSCaT amplitude in individual treatments over time. Vehicle or mAb added after 5 min baseline. **(E)** Normalized mSCaT amplitude over recording on per spine basis. A two-way ANOVA was performed to analyze the effect of mAb and time on mSCaT amplitude. Compared to the control mAb, GluN1 mAb from a patient with anti-NMDAR encephalitis showed a small decrease in Ca^2+^ influx through NMDARs activated by spontaneous glutamate release (spontaneous release) after 10 min exposure. **(F)** Normalized mSCaT amplitude over recording session (mean ± SEM for each group; *n* = 6–7 cells). A two-way ANOVA was performed to analyze the effect of mAb and time on mSCaT amplitude on per cell basis. There was no statistically significant interaction between the effects of mAb and time [*F*(4, 32) = 0.07, *p* = 1.0]. Simple main effects analysis showed that mAb did not have a statistically significant effect on mSCaT amplitude (*p* = 0.9). Simple main effects analysis showed that time did have a statistically significant effect on mSCaT frequency (*p* = 0.001). Both timepoints were significantly increased from baseline using Dunnett’s multiple comparisons test (5–10 min, *p* = 0.0006; 10–15 min, *p* = 0.009). **(G)** Dot plots of normalized mSCaT frequency in individual treatments over time. Vehicle or mAb added after 5 min baseline. **(H)** Normalized mSCaT frequency over recording session. A one-way ANOVA was performed to analyze the effect of mAb on mSCaT frequency at 5–10 min [*F*(2, 2,575) = 4.8, *p* = 0.009] and 10–15 min [*F*(2, 2,575) = 12, *p* ≤ 0.0001] timepoints. Unexpectedly, there was a significant reduction in the mSCaT frequency observed with GluN1 mAb compared to control mAb at 5–10 min (*p* = 0.007, Fisher’s LSD) and 10–15 min (*p* = 0.01, Fisher’s LSD) timepoints. This effect was significant also observed comparing GluN1 mAb to vehicle at 1–5 min (*p* = 0.01, Fisher’s LSD) and 5–10 min (*p* < 0.0001) exposure time. The control mAb had no effect compared to vehicle at 5–10 min (*p* > 0.05, Fisher’s LSD), but reduced mSCaT frequency at 10–15 min compared to vehicle (*p* = 0.03, Fisher’s LSD). **(I)** Normalized mSCaT frequency over recording session (mean ± SEM for each group; *n* = 6–7 cells). A two-way ANOVA was performed to analyze the effect of mAb and time on mSCaT frequency. There was no statistically significant interaction between the effects of mAb and time [*F*(4, 32) = 0.38, *p* = 0.8]. Simple main effects analysis showed that mAb did not have a statistically significant effect on mSCaT frequency (*p* = 0.8). Simple main effects analysis showed that time did have a statistically significant effect on mSCaT frequency (*p* = 0.0012). *Post-hoc* Dunnett’s multiple comparisons test revealed mSCaT frequency at 10–15 min was significantly different from baseline (*p* = 0.0012). Ctrl, control; mAb, human monoclonal antibody; ns, not significant; Veh, vehicle; *p < 0.05, **p < 0.01, ***p < 0.0001; ns, *p* > 0.05.

We adapted a live-cell Ca^2+^ imaging approach using GCaMP6f (Metzbower et al., 2019) to test the effects of human GluN1 antibodies on NMDAR-dependent Ca^2+^ influx at individual synapses. To determine if pathogenic GluN1 antibodies alter Ca^2+^ flux through native synaptic NMDARs, we measured NMDAR-dependent mSCaTs (Figure 4A). We measured baseline mSCaT amplitude and frequency in dendritic spines for 5 min, then applied antibodies or controls and imaged mSCaTs for another 10 min (design shown in Figure 1). We observed a high degree of variability in mSCaT amplitude and frequency at individual spines (Figures 4B,C), consistent with our previous observations (Metzbower et al., 2019). Further, we found that mSCaT amplitude increased over the recording session in all conditions when analyzed on per spine basis (Figure 4D). There was no statistically significant interaction between the effects of mAb and time [*F*(4, 6,748) = 1.1, *p* = 0.3] and simple main effects analysis showed that mAb had a statistically significant effect on mSCaT amplitude on spine basis (*p* = 0.02). The GluN1 mAb did not alter mSCaT amplitude. Overall, we observed a modest decrease in normalized mSCaT amplitude by the GluN1 mAb (1.04 ± 0.022) compared to the control mAb (1.10 ± 0.028, *p* < 0.05, Fisher’s LSD, Figure 4E) when analyzed on a per spine basis at 10–15 min. This effect was also present comparing normalized mSCaT amplitude of GluN1 mAb with vehicle (1.10 ± 0.028, *p* < 0.05, Fisher’s LSD). No significant difference was observed in normalized mSCaT amplitude between control mAb and vehicle. A trend toward reduced normalized mSCaT amplitude at 5–10 min timepoint in GluN1 mAb (1.05 ± 0.021) compared to control mAb (1.11 ± 0.027, *P* = 0.05, Fisher’s LSD) was observed. This trend was evidenced only when individual spine analyses were conducted (Figure 4E). No effect of GluN1 mAb compared to the control mAb or vehicle was observed in mSCaT amplitude when analyzed on per cell basis (Figure 4F), although time similarly influenced amplitude at both 5–10 min and 10–15 min as observed when analyzed per spine. These data suggest that the decreased mSCaT amplitude by GluN1 mAb observed with per spine analysis reflects synapse-to-synapse variation in susceptibility to the GluN1 mAb.

Regarding mSCaT frequency on a per spine basis, we found that there was an increase in all conditions over the recording sessions (Figure 4G). Overall, there was no effect of GluN1 mAb on mSCaT frequency. We observed an interaction between mAb and time on mSCaT frequency (Figure 4H). Unexpectedly, there was a significant reduction in the mSCaT frequency observed with GluN1 mAb compared to control mAb at 5–10 min (*p* = 0.007, Fisher’s LSD) and 10–15 min (*p* = 0.01, Fisher’s LSD) timepoints. This effect was also observed comparing GluN1 mAb to vehicle at 1–5 min (*p* = 0.01, Fisher’s LSD) and 5–10 min (*p* < 0.0001) exposure time (Figure 4H). The control mAb had no effect compared to vehicle at 5–10 min (*p* > 0.05, Fisher’s LSD), but reduced mSCaT frequency at 10–15 min compared to vehicle (*p* = 0.03, Fisher’s LSD). Interestingly, these between group effects on mSCaT frequency are not observed when data were analyzed on a per cell basis (Figure 4I). Simple main effects analysis showed that time had a statistically significant effect on mSCaT frequency (*p* = 0.001). *Post-hoc* Dunnett’s multiple comparisons test revealed mSCaT frequency at 10–15 min was significantly different from baseline (*p* = 0.001). Median mSCaT amplitude and frequency values from per cell basis remained stable over recording sessions (Supplementary Figures 2A,B). These data further support the idea that subsets of synapses are susceptible to transient decrease in mSCaT frequency by GluN1 mAb. Given that the overall normalized mSCaT event number and amplitude were unchanged on a cell-based analysis, the biological relevance of the spine-based analysis observation remains to be defined. Nonetheless, there may be a population of spines that exhibit smaller Ca^2+^ influx in response to glutamate release in the setting of GluN1 Ab exposure. This novel finding supports the notion that pathogenic anti-NMDAR antibodies may alter synaptic NMDAR function after short-term exposure. Further, the effects of pathogenic GluN1 mAbs on presynaptic function warrants further investigation.

## Discussion

We have demonstrated here that an individual human monoclonal antibody, cloned from a patient with anti-NMDAR encephalitis, rapidly localizes to synaptic NMDARs at native hippocampal synapses. We analyzed mSCaTs in cultured hippocampal neurons with the genetically encoded Ca^2+^ indicator GCaMP6f and found that the GluN1 mAb does not alter the amplitude or frequency of synaptic NMDAR-dependent Ca^2+^ influx within 10 min of antibody exposure. There are changes observed between GluN1 mAb compared to control mAb when mSCaTs are analyzed on per spine basis, including changes in mSCaT amplitude and frequency. Surprisingly, these effects are not evident when analyzed on a cell basis, suggesting unique spine features associated with vulnerable NMDARs yet to be discovered. Further, the cause of mSCaT frequency changes, and possible altered presynaptic function, remain undefined. Additionally, we determined by complementary imaging methods that synaptic NMDAR labeling is robust following brief live-cell GluN1 mAb exposure. Further investigation into the dynamics of surface expression of synaptic NMDARs and internalization of GluN1 mAb remain to be examined within this time-frame in cultured hippocampal neurons. These data represent, to our knowledge, the first investigation of an individual anti-GluN1 pathogenic antibody on rapid dysregulation of NMDAR function at native neuronal synapses.

The consequences of pathogenic GluN1 antibodies on Ca^2+^ influx through synaptic NMDARs are potentially widespread as the importance of NMDAR-mediated Ca^2+^ signaling in directing the molecular underpinnings of synaptic plasticity, and learning and memory, are well evidenced (Sabatini et al., 2002; Sanderson and Dell’Acqua, 2011; Lisman et al., 2012). At excitatory glutamatergic synapses, synaptic strength is tuned by alterations to the number or single-channel conductance of ionotropic glutamate receptors, primarily α-amino-hydroxy-5-methyl-4-isoxazolepropionic acid receptors (AMPARs) (Banke et al., 2000, 2001; Lee et al., 2003; Oh et al., 2006; Kristensen et al., 2011; Diering and Huganir, 2018). The NMDAR governs this plasticity through its Ca^2+^ permeability and control of postsynaptic signaling. The level of Ca^2+^ influx through NMDARs at the postsynaptic membrane controls NMDAR-dependent plasticity (Sabatini et al., 2002; Lisman et al., 2012). Also, recent evidence supports a non-canonical metabotropic function of NMDAR in controlling plasticity independent of Ca^2+^ influx (Nabavi et al., 2013; Aow et al., 2015; Dore et al., 2015). Classically, strong activation of NMDARs and a large Ca^2+^ influx induces synaptic strengthening, or LTP, whereas weaker activation NMDAR activation favors long-term depression (LTD). Anti-NMDAR encephalitis antibodies that decrease NMDAR Ca^2+^ influx with receptor activation are likely to disrupt the propensity for synapses to undergo normal LTP, and targeted synapses may exhibit a higher threshold for LTP induction. Further, these targeted synapses may also be more susceptible to LTD following receptor activation. It remains to be explored whether anti-NMDAR antibodies dysregulate metabotropic NMDAR function. In support of the notion of an effect on LTP, CSF from a patient with anti-NMDAR encephalitis has been observed to prevent normal hippocampal NMDAR-LTP at Schaeffer collaterals with high-frequency stimulation after just 5 min of exposure (Zhang et al., 2012). However, serum and CSF in patients with anti-NMDAR encephalitis may contain multiple biologically active factors. As such, synaptic dysfunction mediated by short-term exposure to pathogenic anti-NMDAR CSF is complicated to interpret. There is strong evidence that pathogenic anti-NMDAR antibodies share the same major epitope on the GluN1 subunit (Zhang et al., 2012; Sharma et al., 2018a,b). However, pathogenic anti-NMDAR antibody titers do not correlate with disease severity (Gresa-Arribas et al., 2014) and CSF contains a polyclonal population of pathogenic antibodies that are likely unique to each patient. Also, anti-NMDAR encephalitis is highly heterogenous clinically with regards to disease severity, progression, and response to therapeutics. These features suggest that other antibody parameters such as antibody affinity (Ly et al., 2018) or polyclonal antibody responses, as well as parallel immune responses, may be relevant in disease pathophysiology and disease severity. Further experimentation is required to define the mechanism for rapid altered plasticity induction by anti-NMDAR encephalitis antibodies or factors present in patient CSF.

The pathophysiology underlying anti-NMDAR encephalitis is complex. Internalization of NMDARs by GluN1 antibodies is crosslinking dependent (Hughes et al., 2010), and the effects of pathogenic anti-NMDAR antibodies can be prevented by activation of other synaptic signaling receptors that physically interact with NMDARs (Mikasova et al., 2012; Planagumà et al., 2015, 2016; Washburn et al., 2020). The potential mechanisms by which pathogenic GluN1 antibodies alter NMDAR function aside from receptor internalization are numerous and diverse, including altered channel kinetics by direct allosteric modulation, disruption of extracellular or intracellular binding interactions, altered metabotropic signaling, and altered NMDAR subunit phosphorylation state. Numerous allosteric modulators are known to bind GluN2 NTDs or the GluN1-GluN2 interface and induce changes to single channel properties (Hansen et al., 2010). Allosteric modulators of GluN1-containing NMDARs have only recently been described (Strong et al., 2021). Antibody effects on NMDAR channel function have been explored by single-channel electrophysiology, showing CSF from patients with anti-NMDAR encephalitis increase open time of GluN1/GluN2B receptors in *Xenopus* oocytes (Gleichman et al., 2012). This supports the potential role of pathogenic antibodies as positive allosteric modulators of NMDARs, but these studies have been limited to heterologous expression systems with GluN2B-containing receptors. In contrast, we observed a small decrease in mSCaT amplitude (compared to control mAb) on a spine-based analysis at native synapses, which could be explained by negative allosteric modulation by the GluN1 mAb, possibly through changes to channel open probability, agonist affinity, open time, or Ca^2+^ conductance. The observed change in mSCaT frequency could imply altered presynaptic function, which is challenging to explain *via* postsynaptic NMDAR regulation. Possible mechanisms could include retrograde signaling or other indirect effect on presynaptic function. Also, GluN1 mAb may alter presynaptic NMDAR function, which is known to regulate spontaneous glutamate release (Sjöström et al., 2003; Corlew et al., 2007; Brasier and Feldman, 2008). Nonetheless, this suggests the possibility that individual GluN1 clones within the polyclonal population of patient CSF may differentially modulate NMDAR function. GluN1 antibodies most commonly target an epitope dependent on residues N368/G369 at the “hinge” of the GluN1-NTD clamshell, and their modulation of receptors highlights the importance of binding interactions in this region as NMDAR modulation by EphB receptor binding has been observed at this site as well (Washburn et al., 2020). The heterologous expression approaches lack native receptor complexes and synaptic protein-protein interactions, including those at the GluN1-NTD (Gleichman et al., 2012). Additionally, the native synaptic binding partners modulate NMDAR function (Bard and Groc, 2011) and the GluN2A subunit-containing NMDARs are more prevalent at the synapse than GluN2B subunit-containing NMDARs (Vieira et al., 2020). Further, recent studies have illustrated the regulation of dopamine receptor function by anti-NMDAR antibodies thru NMDAR and D1R crosstalk (Gréa et al., 2019; Carceles-Cordon et al., 2020). Collectively, these studies underscore the complex NMDAR protein-protein interactions that may be targets and altered in anti-NMDAR encephalitis.

An additional mechanism by which the GluN1 mAb may alter mSCaT amplitude is through disruption of extracellular synaptic binding partners and increased lateral mobilization of surface receptors. Antibody-mediated internalization of NMDARs by pathogenic patient CSF was found to be prevented by activation of EphB2 receptors *in vitro* and *in vivo* (Mikasova et al., 2012). The exact mechanism underlying this regulation remains to be defined in anti-NMDAR encephalitis. However, the working hypotheses include altered synaptic capture, receptor stabilization, and receptor mobility. Patient CSF also produced increased mobility of GluN2A-containing receptors and decreased mobility of GluN2B-containing receptors, which was also prevented by activation of EphB2 receptors. Although this was not evaluated on the same timescale in which we observed decreased mSCaT amplitude, that study used patient CSF while we studied an individual GluN1 mAb that may have distinct effects. EphB receptors regulate synaptic NMDAR function and targeting (Henderson et al., 2001; Nolt et al., 2011), and it is very possible that pathogenic GluN1 antibodies alter NMDAR surface trafficking by disrupting binding between these two receptors at their nearby site of interaction. Such an effect would alter NMDAR mobility and diffusion, potentially away from site of spontaneous release of glutamate as measured by our mSCaT assay here. Because NMDARs activated by spontaneous release likely represent a population of receptors at least partially non-overlapping with those activated by evoked release, their localization across from the site of spontaneous release may be critical for maximum post-synaptic response and Ca^2+^ influx. Trafficking of NMDARs away from sites of spontaneous vesicle release therefore may be an underlying mechanism by which the GluN1 mAb may reduce mSCaT amplitude.

This study involved time-resolved and spatially restricted analysis of NMDAR function at hippocampal synapses. As such, it will be challenging to extend these observations to biochemical metrics of GluN1 mAb function. Further, we characterized a single GluN1 mAb, clone 5F5, in these studies. Future studies should explore whether the effects described here are reproduced with other GluN1 clones, mixtures of clones, patient CSF, or pathogenic antibodies isolated from patient serum.

Effects specific to spontaneously activated NMDARs, as measured in this study, may be relevant to the underlying pathophysiology of anti-NMDAR encephalitis. Spontaneously activated NMDARs mediate synaptic homeostasis (Reese and Kavalali, 2015) and blockade of spontaneous release leads to potentiation of synaptic transmission (Nosyreva et al., 2013). Both phenomena may be dysregulated in anti-NMDAR encephalitis. However, it is important to consider that evidence suggests NMDARs activated by spontaneous release may be distinct from those activated by neurotransmitter release following action potentials (Atasoy et al., 2008). Further examination of short-term effects of GluN1 mAb on action potential-evoked NMDAR Ca^2+^ transients is therefore necessary to define its effect on these distinct synaptic NMDAR populations. Future experiments will further interrogate these hypotheses about the underlying pathophysiology of anti-NMDAR encephalitis by evaluating GluN1 antibody effects on native NMDAR function and downstream intracellular signaling pathways. These studies will provide valuable insights into the role of antibody repertoire in the underlying disease pathogenesis and contribute to the foundation for targeted therapeutics and precision medicine in autoimmune encephalitis.

## Data Availability Statement

The raw data supporting the conclusions of this article will be made available by the authors, without undue reservation.

## Ethics Statement

The animal study was reviewed and approved by the Institutional Animal Care and Use Committee at University of Maryland School of Medicine.

## Author Contributions

CD designed the research, performed the experiments, analyzed the data, and drafted the manuscript. SM contributed to the analytic tools and analyzed the data. SD provided the human mAb reagents. TB designed the research. DB designed the research, analyzed the data, and revised the manuscript. All authors contributed to the article and approved the submitted version.

## Conflict of Interest

The authors declare that the research was conducted in the absence of any commercial or financial relationships that could be construed as a potential conflict of interest.

## Publisher’s Note

All claims expressed in this article are solely those of the authors and do not necessarily represent those of their affiliated organizations, or those of the publisher, the editors and the reviewers. Any product that may be evaluated in this article, or claim that may be made by its manufacturer, is not guaranteed or endorsed by the publisher.

## Supplementary Material

Supplementary Figure 1 Basal calcium levels at synaptic spines analysis on per cell basis. Dot plots of normalized basal F values in individual treatments over time per cell. Vehicle or mAb added after 5 min baseline. Note the F values remained stable following addition of vehicle or mAbs when analyzed on per cell basis. Ctrl, control; mAb, human monoclonal antibody; Veh, vehicle.

Supplementary Figure 2 mSCaT amplitude and frequency analyses on per cell basis. **(A)** Dot plots of normalized mSCaT amplitude in individual treatments over time per cell. Vehicle or mAb added after 5 min baseline. **(B)** Dot plots of normalized mSCaT frequency in individual treatments over time.
