# Supplementary figures and images for: Regulation of NMDA Receptor Signaling at Single Synapses by Human Anti-NMDA Receptor Antibodies
Source: Front Mol Neurosci. 2022 Jul 28;15:940005. doi: 10.3389/fnmol.2022.940005 (PMC9371948; doi:10.3389/fnmol.2022.940005)

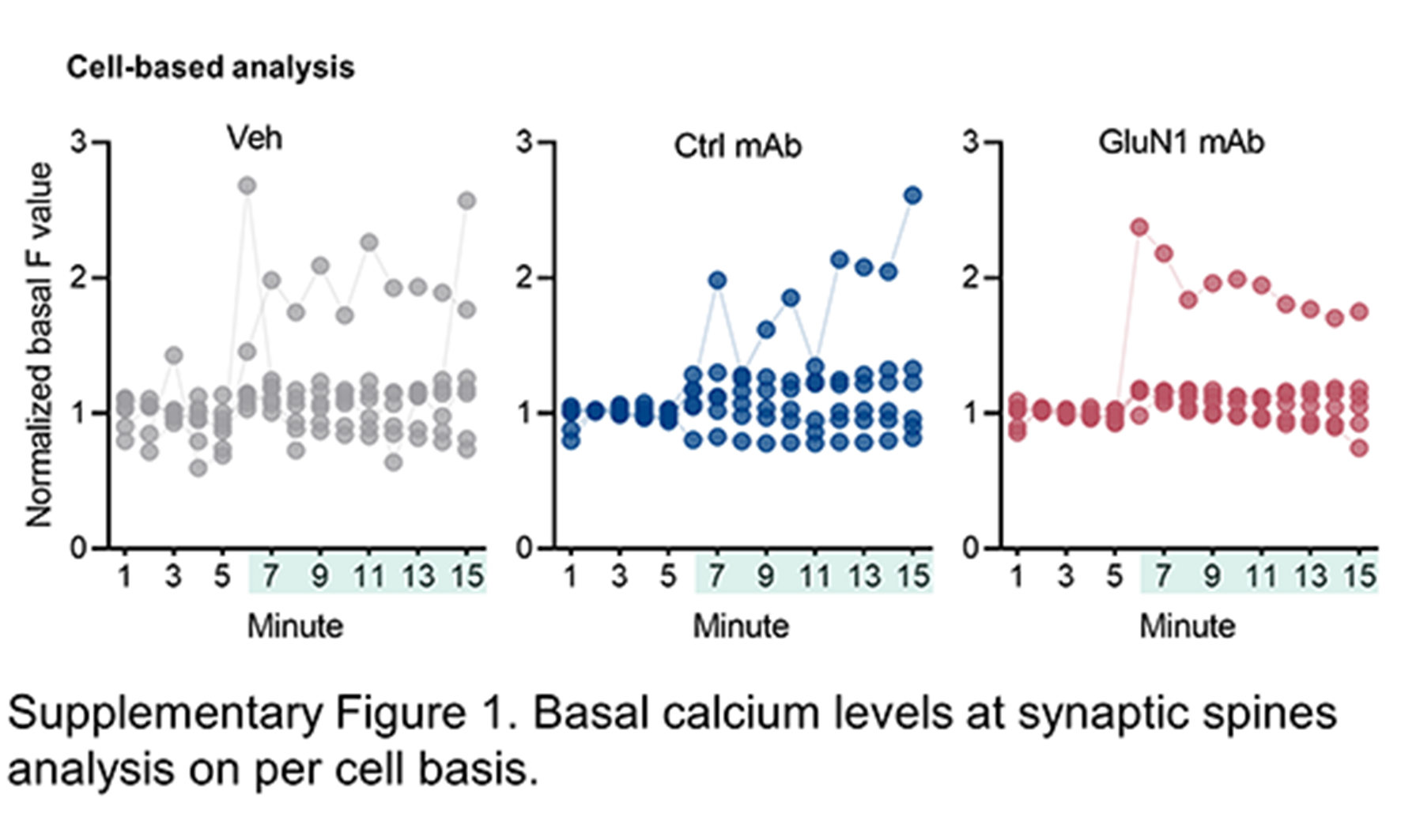

Supplement: Supplementary Figure 1 — Basal calcium levels at synaptic spines analysis on per cell basis. Dot plots of normalized basal F values in individual treatments over time per cell. Vehicle or mAb added after 5 min baseline. Note the F values remained stable following addition of vehicle or mAbs when analyzed on per cell basis. Ctrl, control; mAb, human monoclonal antibody; Veh, vehicle. [file Image_1.tif]

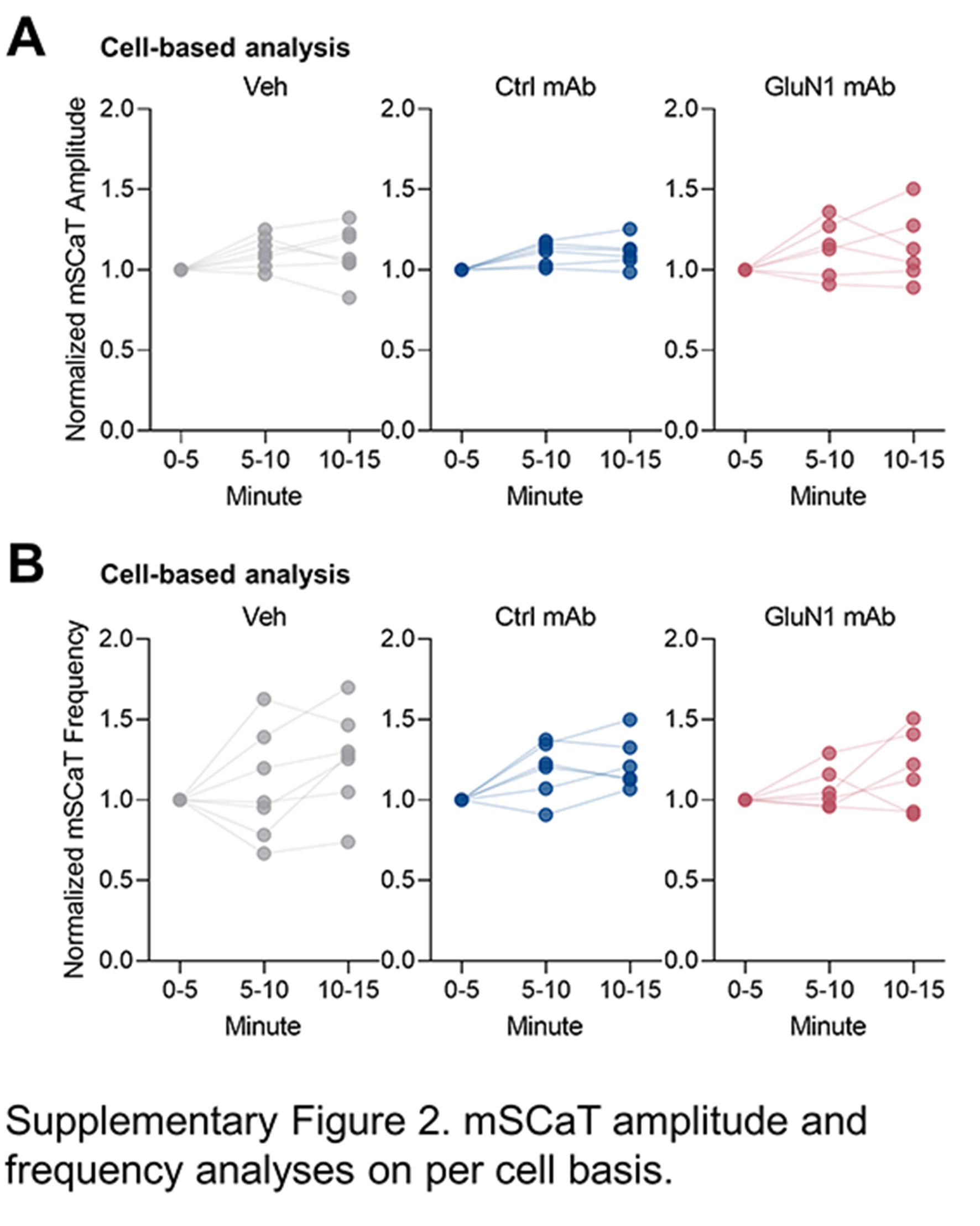

Supplement: Supplementary Figure 2 — mSCaT amplitude and frequency analyses on per cell basis. (A) Dot plots of normalized mSCaT amplitude in individual treatments over time per cell. Vehicle or mAb added after 5 min baseline. (B) Dot plots of normalized mSCaT frequency in individual treatments over time. [file Image_2.tif]
